# Potential Molecular Mechanisms of Recurrent and Progressive Meningiomas: A Review of the Latest Literature

**DOI:** 10.3389/fonc.2022.850463

**Published:** 2022-05-30

**Authors:** Wenjie Peng, Pei Wu, Minghao Yuan, Bo Yuan, Lian Zhu, Jiesong Zhou, Qian Li

**Affiliations:** ^1^ Department of Pediatrics, Army Medical Center, Army Medical University, Chongqing, China; ^2^ Department of Neurosurgery, The First Affiliated Hospital of Harbin Medical University, Harbin, China; ^3^ Department of Neurology, Chongqing Medical University, Chongqing, China; ^4^ Department of Nephrology, The Dazu District People’s Hospital, Chongqing, China; ^5^ Department of Plastic Surgery, Changhai Hospital Affiliated to Naval Medical University, Shanghai, China

**Keywords:** meningioma, mechanisms, cell proliferation, neo-angiogenesis, apoptosis, immunogenicity, genetic alterations

## Abstract

Meningiomas, the most frequent primary intracranial tumors of the central nervous system in adults, originate from the meninges and meningeal spaces. Surgical resection and adjuvant radiation are considered the preferred treatment options. Although most meningiomas are benign and slow-growing, some patients suffer from tumor recurrence and disease progression, eventually resulting in poorer clinical outcomes, including malignant transformation and death. It is thus crucial to identify these “high-risk” tumors early; this requires an in-depth understanding of the molecular and genetic alterations, thereby providing a theoretical foundation for establishing personalized and precise treatment in the future. Here, we review the most up-to-date knowledge of the cellular biological alterations involved in the progression of meningiomas, including cell proliferation, neo-angiogenesis, inhibition of apoptosis, and immunogenicity. Focused genetic alterations, including chromosomal abnormalities and DNA methylation patterns, are summarized and discussed in detail. We also present latest therapeutic targets and clinical trials for meningiomas' treatment. A further understanding of cellular biological and genetic alterations will provide new prospects for the accurate screening and treatment of recurrent and progressive meningiomas.

## Introduction

Meningiomas generally originate from the meninges and meningeal spaces. They are the most frequently occurring primary intracranial tumors of the central nervous system in adults, with an incidence of 7.86 cases per 100,000 people every year (1). According to the 2021 World Health Organization (WHO) tumor classification, meningiomas are classified as benign (>80%), atypical (15%–20%), and anaplastic (1.0%–3.0%), depending on the mitotic rate, brain invasion, or specific histological features (2–4). Although the majority (~80%) of meningiomas are benign and could be cured or become stable through surgical resection, some present with high-risk behaviors and poor prognosis, including early or high-rate recurrence and rapidly progressive course even after radiotherapy (5). More interestingly, among meningiomas with benign pathological features, 7–25% histologically tend to relapse or become malignant after surgical resection (6). Atypical and anaplastic meningiomas are naturally substantially more aggressive, and their recurrence rates in 5 years reach up to 30–50% and 90%, respectively (7, 8). Radiotherapy is recommended for partially resected Grade II and all Grade III meningiomas. Nevertheless, a subset of patients with Grade II meningiomas may live through a benign clinical course with no need for radiotherapy (9). The histological grade does not fully reflect the biological behavior of meningiomas to currently guide treatment. Hence, there is a need to explore useful predictors of the clinical behavior or overall prognosis of meningiomas.

Previous studies have shown that the risk factors of meningiomas are complex, including age, sex, radiation, trauma, diabetes mellitus, and arterial hypertension (1, 7), and the progression of recurrent meningiomas involves numerous factors, including Simpson grade IV/V resection, a larger tumor size, tumor location, high vascular endothelial growth factor receptor (VEGFR) expression, WHO Grade II/III, high Ki-67 expression, and lack of progesterone receptor expression (10). Recurrent meningiomas may be accompanied with malignant transformation and multiple treatments or limited optional drugs, making management much more challenging (7, 11). Therefore, a further understanding of the molecular mechanisms underlying the recurrence or progression will help predict the clinical behavior, which is beneficial for early recognition of high-risk meningiomas and timely adjustment of treatment protocols.

In addition to the traditional WHO grading, the latest studies on meningiomas have provided insights into the genomic alterations, including DNA somatic copy number, DNA point mutation, DNA methylation, and transcriptomic and proteomic data (12). Advances in molecular classification through DNA methylation have gradually been approved by researchers (1, 6, 9, 12, 13). Similar to other central nervous system neoplasms, such as glioma, Nassiri reported that meningiomas could be classified into different molecular groups with distinct and prototypical biological features after a comprehensive analysis combining copy number, DNA methylation, and mRNA sequencing data (12), complementing existing WHO grades. Here, based on the most up-to-date biomedical research knowledge, we review the potential cellular biological mechanisms and molecules involved in the recurrence or progression of meningiomas from several perspectives, including the excessive proliferation of tumor cells, neo-angiogenesis, inhibition of apoptosis, immunogenicity, and genetic alterations involving chromosomes and genes related to meningiomas (3, 13). Further, we summarize existing therapeutic targets and clinical trials for meningiomas’ treatment. We expect this information to allow for an exploration of more accurate prognostic markers and potential targeted therapies for meningiomas.

## Cell Proliferation

**Figure 1:**
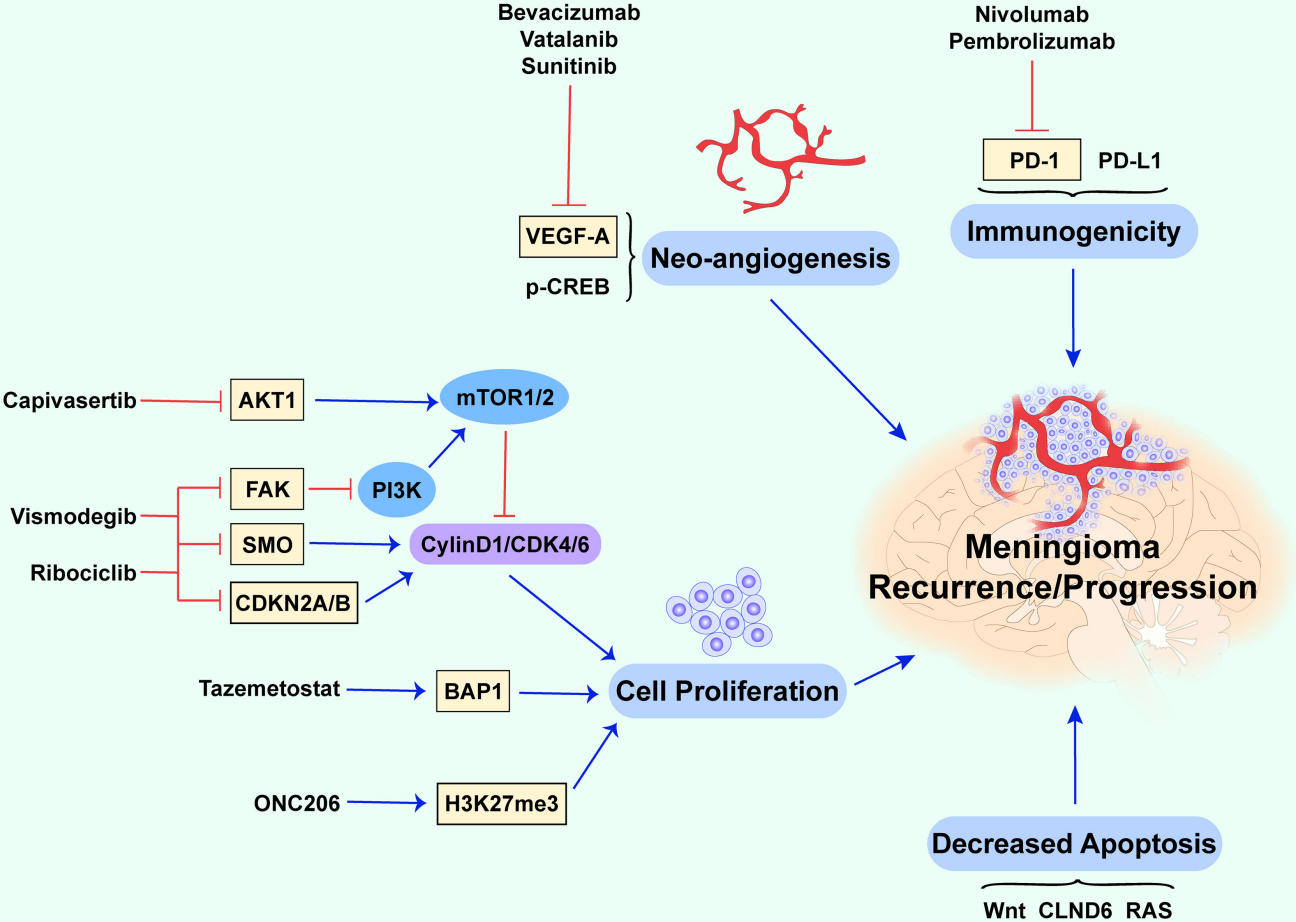
Potential biological mechanisms of recurrent and progressive meningiomas. This figure briefly summarizes several cellular biological mechanisms and molecules contributing to recurrent and progressive meningiomas. The abnormal proliferation of tumor cells, downregulation of apoptotic processes, neo-angiogenesis, and immunogenicity together promote recurrence and progression (the red lines show an inhibitory effect and the blue arrows show a promoting effect).

Recurrent or progressive meningiomas usually begin with excessive cell growth and proliferation. Evidence suggests that tumor cell growth and proliferation are tightly linked to cell-cycle dysregulation (4). Disordered cell-cycle proteins, the uncontrolled regulation of transcription factors, and mutations in cell-cycle-related genes can promote cell proliferation and differentiation in meningioma (14–17). The cell-cycle-related proteins topoisomerase IIα and mitosin, which play important roles in regulating mitotic chromosome condensation and separation (18), are positively associated with a high risk of meningioma recurrence (15). Forkhead box protein M1 (FOXM1), a master transcription factor for cell growth and proliferation, is closely associated with hepatocellular carcinoma (19), prostate cancer (20), glioma (21), and basal cell carcinoma (22). FOXM1 is thought to accelerate G1/S and G2/M transition to promote mitotic progression (14). A recent comprehensive molecular profiling study indicated that the expression of FOXM1 is relevant to increased proliferation and poor clinical prognosis (23). Similarly, the results obtained in a newly established model of meningioma showed that FOXM1 overexpression increases proliferation in benign meningioma, whereas its depletion decreases proliferation in malignant meningioma (24). As such, thiostrepton, a FOXM1 inhibitor, combined with radiation therapy, was found to noticeably prevent the proliferation of malignant meningioma cells **(** **Figure 1** **)** (24).

Gene mutations in v-Akt murine thymoma viral oncogene (*AKT1*), homolog 1 smoothened, frizzled class receptor (*SMO*), focal adhesion kinase (*FAK*), cyclin-dependent kinase inhibitor 2A/B (*CDKN2A/B*), and dystrophin-encoding and muscular dystrophy-associated (*DMD*) are also considered to be associated with cell proliferation in meningioma (17). *AKT1* encodes the AKT1 kinase (a serine/threonine-protein kinase), and the overactivation of AKT1 can lead to uncontrolled cell growth and proliferation *via* the phosphatidylinositol 3-kinase (PI3K)/AKT/mammalian target of rapamycin (mTOR) signaling pathway **(** **Figure 1** **)** (25, 26). mTOR, mainly regulated by the PI3K/AKT pathway, is highly expressed in various tumors and is closely associated with cell growth and proliferation (17). Studies have indicated that the overactivation of mTOR results in a high mitotic index (27) and contributes to the recurrence of meningioma (28) and poorer outcomes (27).

*SMO* mutations lead to cell-specific proliferation and mediate the development of meningioma through uncontrolled activation of the sonic hedgehog signaling pathway (25, 29, 30). *FAK*, which encodes a cytoplasmic protein tyrosine kinase that mediates cell growth, proliferation, and survival, is overexpressed in some meningiomas (31). Ribociclib, a cyclin-dependent kinase (CDK) inhibitor, was evaluated for its effect on other highly mutated genes (other than the common *NF2*), such as *AKT1* and *SMO* (NCT02933736) (**Table 1**) (38). Moreover, a national Alliance-sponsored cooperative group phase II clinical trial evaluated the efficacy of SMO, AKT1, and FAK inhibitors for recurrent or progressive meningiomas with targetable alterations in SMO, AKT1, and NF2, respectively (NCT02523014/A071401) (5). Vismodegib, included in an ongoing Alliance clinical trial, is a hedgehog pathway-targeting agent tested for SMO/PTCH1-mutated progressive/recurrent meningiomas (NCT02523014) (32).

**Table 1:** Summarization of key molecules and potential targeted therapy in recurrent and progressive meningiomas.

	Targets	Inhibitors	Ongoing Clinical Trial	Reference
**Cell proliferation**	AKT1	Capivasertib Ribociclib	NCT02523014 NCT02933736	(5, 32)
	SMO	Vismodegib Ribociclib	NCT02523014 NCT02933736	(5, 32)
	FAK	Vismodegib	NCT02523014	(5)
	CDKN2A/B	Ribociclib	NCT02933736	(32)
	BAP1	Tazemetostat	NCT02860286	(33)
	H3K27me3	ONC206	NCT04541082	(34)
**Neo-angiogenesis**	VEGF-A	Bevacizumab Vatalanib Sunitinib Apatinib mesylate Erlotinib hydrochloride	NCT01125046 NCT00348790 NCT00589784 NCT04501705 NCT00045110	(35, 36)
**Immunogenicity**	PD-1	Nivolumab Pembrolizumab	NCT02648997 NCT04659811 NCT03279692	(37)
**Chromosomal abnormalities**	NF2 and/or SMARCB1	Everolimus Vistusertib Dasatinib Selumetinib	NCT00972335 NCT03095248 NCT00788125 NCT03095248	(5, 35, 38–40)

*CDKN2A* encodes p16INK4A and p14ARF, and *CDKN2B* encodes p15INK4B. p15INK4B and p16INK4A prevent S-phase entry by inhibiting the CDK4/cyclin D complex and are generally mutated in Grade II and III meningiomas. p14ARF prevents cell proliferation in the G1 phase and decelerates p53 degradation through downregulation of the proto-oncogene murine double minute 2 protein (*MDM2*) (41). The mutation or deletion of *CDKN2A* and *CDKN2B* has been linked to a poorer prognosis in meningioma (42). A CDK inhibitor combined with ribociclib could be a potential treatment approach for meningiomas with mutations in the tumor-suppressor genes *CDKN2A* and *CDKN2B* (NCT02933736) (32). Moreover, mutations in the tumor-suppressor gene *p53* also affect the occurrence and development of meningioma (43). When mutations occur, p53 changes from a tumor suppressor to a tumor promoter owing to structural changes that suppress its roles in inhibiting cell growth and apoptosis, leading to cancer (44). The p53 mutation rate is higher in atypical and malignant meningiomas, and most importantly, it is higher in recurrent than in non-recurrent diseases (45). Some researchers also found that the combination of p53 and Ki67 could be a promising predictor of recurrence in meningiomas (45). *DMD* encodes dystrophin, which regulates cytoskeleton remodeling and cell proliferation in response to extracellular signal stimulation (46, 47). The deletion of *DMD* contributes to progressive meningioma and a shorter overall survival (16), partly due to the defective inhibition of cell proliferation leading to disease progression (48). Breast cancer 1-associated protein-1 (BAP1), a deubiquitylating enzyme, is a tumor suppressor. Familial and sporadic BAP1-deficient meningiomas tend to be rare, and aggressive malignant tumors (grade III) are associated with increased aggressiveness and poorer prognosis (49). Tazemetostat, a BAP1 inhibitor, increases the level of the PCR2 complex protein EZH2, activated by BAP1, and might be a potential drug for rhabdoid meningioma caused by BAP1 loss (NCT02860286) (50).

In addition to the genes mentioned previously herein, a 2018 study showed that mutations in the promoter of telomerase reverse transcriptase (*TERTp*) enhance the degree of malignancy of meningiomas and lead to poor prognosis (51). Other studies have corroborated that *TERTp* mutations predict poor survival in progressive/high-grade meningiomas (33, 52, 53). Telomere maintenance is a marker of tumor formation, and most tumors express telomerase to prevent telomere shortening (52). Telomerase activation caused by *TERTp* mutations enforces cell immortalization and promotes the growth of tumors (51), which could be observed in recurrent and malignant tumors (54). Furthermore, a 2021 study revealed that *TERT* alterations are a biomarker of meningioma progression and reduce progression-free survival after adjuvant radiotherapy (55). Hence, we suggest that *TERTp* mutations can significantly predict poor prognosis in meningiomas, but no effective targeted drugs have been found to date.

In recent years, it has been reported that the loss of H3K27 trimethylation (H3K27me3) plays a prominent role in the recurrence of meningioma (56). Further research found that the loss of H3K27me3 predicts early recurrence and death for grade 2, but not for grade 3, meningioma (57). H3K27me3 affects DNA damage repair and contributes to several biological processes, including cell differentiation, proliferation, and stem-cell plasticity (58). The latest study found that ONC206, a DRD2 antagonist and ClpP agonist, is orally bioavailable, penetrates the blood-brain barrier, and exhibits anti-cancer efficacy without toxicity, and it is currently the subject of an ongoing trial (NCT04541082) for H3K27M-mutant, malignant meningiomas and other central nervous system tumors (34). However, its therapeutic effect on tumorigenesis or cancer recurrence with respect to H3K27me3 requires further clinical trials.

## Neo-Angiogenesis

Neo-angiogenesis is one of the most important features of higher-grade meningiomas. On the one hand, it makes the tumor grow rapidly, and on the other hand, it makes surgical resection more difficult based on the rich blood supply. Tumor vessel density is a key feature during oncogenesis and is tightly correlated with the upregulation of vascular endothelial growth factor (VEGF), placental growth factor, and insulin-like growth factor-binding protein-3 (59). A recent follow-up study investigated VEGF and its three receptors in meningiomas and demonstrated a significant increase in VEGF-A levels in WHO grade III meningiomas (60). VEGF-A, an endothelial cell-specific mitogen, contributes to new blood vessel growth (35, 61). Upon overexpression, VEGF-A contributes to the rapid growth of tumors (35) and regulates maturation and stabilization during the late stages of tumors (62). VEGF-A is a powerful mitogenic and angiogenic disulfide-linked homodimer, which is secreted from tumors and increased under conditions of ischemia for the rapid expansion of tumor vessels (63). VEGF-A is tightly associated with refractory or higher-grade meningiomas (35), becoming a potential therapeutic target with the foundation of anti-angiogenic agents blocking the VEGF pathway (64). Antiangiogenic drugs, such as bevacizumab, vatalanib, and sunitinib, were reported to reduce the recurrence rate of meningiomas significantly (1). Two prospective phase II trials involving patients with refractory meningiomas have evaluated the efficacy and safety of bevacizumab (36). One study of 40 patients treated with bevacizumab indicated that the progression-free survival (PFS) at 6 months is 87% for grade I meningiomas, 77% for grade II meningiomas, and 46% for grade III meningiomas (NCT01125046). Another clinical trial in 2016 combining bevacizumab with everolimus found a median PFS of 22 months for those with recurrent and progressive meningiomas after surgery and/or radiation therapy (95% CI 4.5–26.8). This combination could block disease progression in 88% of patients (NCT00972335) (35, 36). Both vatalanib (NCT00348790) and sunitinib (NCT00589784) are tyrosine kinase inhibitors targeting VEGFR and were shown to act partly on recurrent meningiomas. Furthermore, other trials like those for apatinib mesylate (NCT04501705) and erlotinib hydrochloride (NCT00045110) found on ClinicalTrials.gov have demonstrated that VEGFR is an emerging therapeutic target.

In addition to VEGF-A, phosphorylated cyclic-AMP responsive element-binding protein (p-CREB) is a novel high-risk molecule abundantly expressed in the endothelia of tumor vessels in all meningiomas, and high p-CREB levels are closely associated with the recurrence of meningiomas (65). p-CREB was found in various tumors, including glioma, because of its physical properties, including binding to upstream signaling kinases and downstream genes (66). It is a transcription factor that participates in numerous cellular processes and induces VEGF expression, leading to neo-angiogenesis in meningiomas (65). Barresi et al. reported that p-CREB expression can be identified in tumor vessels but disappears in the vessels of the normal adult and neonatal leptomeninges, implying that p-CREB is related to neo-angiogenesis (65). The relationship between p-CREB and VEGF has not been fully elucidated and requires further study.

The levels of inflammation in perivascular areas of the tumor, induced by ischemia or other proteins, can also affect the neo-angiogenesis of meningiomas. VEGF-A is a downstream target of hypoxia-inducible factor 1α (HIF-1α), a molecular marker of hypoxia (67). A large cohort study of 263 patients with meningiomas found that upregulated levels of HIF-1α and VEGF-A could significantly predict the recurrence of meningiomas (68). Moreover, HIF-1α and VEGF-A are correlated with peritumoral edema (69), which was demonstrated to be associated with poor prognosis in meningiomas.

## Resistance to Apoptosis

Apoptosis, a well-known form of cell death that occurs in response to external stimuli or internal stresses, is generally inhibited in tumor cells, resulting in uncontrolled proliferation (70). Numerous studies have indicated that the Wnt signaling pathway has an important role in resistance to apoptosis in neurological disorders, such as stroke (71, 72), spinal cord injury (73, 74), neuroblastoma (75), and glioma (76). The Wnt signaling pathway was recently reported to be associated with the apoptosis of meningioma cells *via* three pathways, the classical Wnt/β-catenin signaling, the planar cell polarity pathway, and the Wnt-Ca^2+^ pathway (77–80). Inhibition of the Wnt/β-catenin pathway by plant medicines leads to apoptosis in human meningioma cells (81). The long non-coding (lnc) RNA SNHG1 was found to inhibit apoptosis in BEN-1-1 and IOMM-Lee cells, and SNHG1 deficiency restrains cell growth and accelerates apoptosis in meningioma cell lines *via* the Wnt pathway (79). Moreover, downregulation of the lncRNA LINC00702 reportedly inhibits Wnt activity and induces apoptosis in malignant meningioma (78). Thus, the Wnt pathway seems to play a negative regulatory role in the apoptosis of meningioma cells; however, the precise underlying mechanism remains unclear.

Recent studies have reported several potential mechanisms of resistance to apoptosis in meningioma. CD163 is a type I membrane protein, the overexpression of which leads to reduced apoptosis in human meningioma cells (82). CLND6, also called claudin6, is a component of tight junctions that contributes to maintaining cell–cell junctions in epithelial cells (83). The downregulation of CLND6 has been associated with tumor occurrence, and its overexpression accelerates apoptosis in cancer cells (84–87). Additionally, CLND6 has been found to regulate migration and invasion capacities in malignant meningioma cell models (88). Rat sarcoma (RAS) is a member of the small GTPase family that participates in the regulation of embryonic development, differentiation, cell-cycle progression, and cell survival (89). The downregulation of RAS activity leads to significantly reduced ERK and AKT phosphorylation, suppresses proliferation, and induces the apoptosis of human meningioma cells (88). Furthermore, let-7d, a member of the let-7 family, has been regarded as a tumor suppressor in various cancers (90–92). Let-7d promotes apoptosis and suppresses the proliferation of meningioma by targeting AEG-1 (93). Based on a genomics analysis of 300 meningiomas, Clark et al. reported that mutations in TNF receptor-associated factor 7 (TRAF7) are also common, and they identified the accumulation of mutations in Krüppel-like factor 4 (KLF4), AKT1, and SMO (29). TRAF7, a pro-apoptotic protein containing an N-terminal RING finger domain, an adjacent TRAF-type zinc finger domain, a coiled-coil domain, and seven C-terminal WD40 repeats, affects several signaling pathways, including the NF-κB pathway, and the ubiquitination of proteins, such as c-FLIP (29, 94). TRAF7 is usually mutated together with KLF4, AKT1, or the phosphatidylinositol-4, 5-diphosphate 3-kinase catalytic subunit α protein (PIK3CA) (95, 96). KLF4 is a transcription factor. AKT1 activates the PI3K/mTOR pathway (96). In 2016, mutations in PIK3CA were found to be frequent drivers of certain meningiomas (97). Mutations in TRAF7, KLF4, AKT1, or PIK3CA are commonly associated with grade 1 meningioma, whereas combined mutations might be associated with a high recurrence rate (98). Therefore, therapies targeting the pro-apoptotic roles in recurrence and malignancy *via* different approaches might contribute to improved prognosis.

## Immunogenicity

Subsets of patients still experience a progressive clinical course even after surgery and radiation, because tumors can evade the immune system *via* certain mechanisms, leading to the formation of an immunosuppressive tumor microenvironment, including the upregulation of programmed death-1 (PD-L1), suppressive cells, such as regulatory T cells, or other unknown proteins (99). Nassiri found that meningiomas are immunogenic, characterized by massive immune infiltration and pertinent pathways including immune regulation and signaling (12). The proteins associated with immune regulation include IL-1, TNF, ING-α, and PD-1. Depending on these data, immunotherapy could be another treatment for these malignant meningiomas (99, 100). NF2 mutations and the loss of chromosome 22 are frequently observed in these meningiomas (12). Therefore, Yeung et al. explored the immunological landscape of meningiomas in an NF2-mutant murine meningioma model and found that these tumors were heavily infiltrated by anti-inflammatory M2 macrophages. Intervention with an anti-CSF1/CSF1R antibody was found to normalize the tumor microenvironment, indicating that targeting the CSF1/CSF1R axis might be a potential treatment for malignant meningiomas (99). PD-1 and PD-L1 are closely associated with higher-grade meningiomas. PD-1 inhibitors, such as nivolumab (NCT02648997) and pembrolizumab (NCT04659811), have a significant effect on preventing the recurrence of meningiomas. A phase II study in 2022 showed that pembrolizumab exerts promising efficacy on a subset of recurrent and progressive grade 2 and 3 meningiomas (NCT03279692). This study reported a lower PFS-6 rate of 0.48 and median PFS of 7.6 months for higher-grade meningiomas compared to those in previous studies (37). Masaki summarized several clinical trials investigating whether PD-1 might affect recurrent meningiomas (100). In addition, the effect of IFN-α was demonstrated in several highly vascularized tumors, such as gliomas and meningiomas (32). A clinical trial of IFN-α-2B found that it could improve the prognosis of grade I recurrent meningiomas and induce disease stability (101).

## Chromosomal Abnormalities

Epigenomics studies have revealed that transcriptional and epigenomic regulatory mechanisms occupy an important part in recurrent and progressive meningiomas. Except for gene mutations and some special molecules, chromosomal abnormalities have been the hot topics these years. It was reported that higher rates of copy-number alterations and karyotypic abnormalities are linked to higher-grade meningiomas (9). Chromosome 22 is the most commonly reported abnormal chromosome in meningiomas. It shows alterations in more than half of meningiomas, especially in benign tumors, with a large proportion of deletions of chromosome 22 occurring in the neurofibromatosis type 2 gene (*NF2*) region, which contributes to the development of meningiomas (4, 102). NF2 promotes contact inhibition and tumor suppression by inhibiting mitotic signaling in the cell cortex (90). However, another study suggested that NF2 might not be involved in meningioma progression (103). A study including 775 samples revealed that the loss of *NF2* or co-occurrence with recurrent *SMARCB1* mutations frequently occurs in atypical meningiomas. *SMARCB1*, located on chromosome 22, might induce the progression of meningiomas. In addition, *NF2* alterations combined with abnormalities in AKT1 and mTOR are associated with the overgrowth of various tissues, which could be responsible for the recurrence of meningiomas (104).

Strong expression of SSTR2A receptors, inhibition of the osteoglycin/mTOR pathway, and activation of NF2 signaling promote apoptosis in malignant meningioma cells (39, 105). A recent phase II CEVOREM trial showed that the combination of everolimus, an mTOR inhibitor, and octreotide, a somatostatin agonist, has an antiproliferative effect on meningiomas (NCT00972335) (39). Atypical *NF2* mutants demonstrate chromosomal instability, which might be related to tumor invasiveness (106). Brigatinib, an inhibitor of multiple tyrosine kinases, was capable of stopping the growth of NF2-deficient xenograft meningiomas for the inhibition of multiple tyrosine kinases, including EphA2, Fer, and focal adhesion kinase 1 (FAK1) (107). A FAK inhibitor (GSK2256098) was identified to significantly improve the survival rates of patients with recurrent or progressive NF2-mutated meningiomas (NCT02933736) (108). Further, a phase II trial revealed another MEK1/2 inhibitor, selumetinib, to have an effect on NF2-related meningiomas (NCT03095248) (5, 38). These advances on NF2-related meningiomas represent a major step forward in therapeutics.

Except for chromosome 22, loss of chromosome 1p was related to recurrent meningiomas, despite total resection and was involved in the activation of the cell cycle (23, 109). Further, the loss of chromosome 14q and complex karyotypes (multiple chromosome mutations) have also been reported as independent recurrence-specific prognostic indicators of meningiomas or malignancy development (103, 106, 110–112). The genes located on chromosome 1p include TP73, CDKN2C, RAD54, EPB41, GADD45A, and ALPL, while the genes inactivated on chromosome 14q are NDRG family member 2 and maternally expressed gene 3 (113). Moreover, the loss of chromosomes 9p, 6q, 10, and 18q or the abnormal gain of 1q, 9q, 12q, 15q, 17q, and 20q has been recently reported (17, 96, 114).

## DNA Methylation Patterns

In addition to those on the aforementioned mutations, studies have been conducted in recent years to understand genetic and epigenetic alterations in meningiomas. Researchers have attempted to compare meningioma grading with DNA methylation classification (MC) (6). Sahm et al. distinguished six methylation classes among 479 patients and found that these classes might predict more current clinical courses than histology (115). DNA MC could finally prove superior to traditional light microscopy in distinguishing recurrent or progressive meningiomas. The DNA MC was divided into two major epigenetic groups, including six subclasses, MC benign 1–3, MC intermediate A and B, and MC malignant, which did not correspond exactly to the WHO grade. Interestingly, most *NF2* mutations were observed in MC benign-1 meningiomas, and other *NF2* mutations were scattered in the different groups. Mutations in four main genes, *AKT1*, *SMO*, *KLF4*, and *TRAF7*, were identified in MC benign-2 tumors. The frequency of CDKN2A and TERT mutations was higher in MC intermediate-B and MC malignant groups. MC benign-1 was related to the loss of chromosome 22q, MC benign-3 was related to frequent mutations in chromosome 5, and MC intermediates A/B and MC malignant were related to the loss of chromosome 1p. The loss of chromosome 22q was related to MC intermediate-B and MC malignant (116) **(** **Table 2** **)**. All of these mutations were found to be tightly associated with the recurrent or progressive meningiomas described previously herein, proving that DNA methylation patterns are helpful for the risk stratification of meningiomas. The authors also showed that DNA methylation patterns provide a more precise prediction of progression-free survival outcomes at 10 years of follow-up than does WHO grading. The predictive power of single or combined DNA MCs was determined to be stronger than that of WHO grades, especially for meningiomas with a WHO grade I histology and patients at a lower risk of recurrence among WHO grade II meningiomas (p = 0.0096) from the Brier prediction test (115). For those genes presenting with mutations associated with any clinical courses, such as *NF2*, DNA methylation guides further risk stratification compared to that with whole-genome sequencing only. Nassiri et al. also found that DNA methylation, combined with clinical factors, leads to a reliable individualized estimation of the 5-year recurrence risk of meningiomas (40). Moreover, DNA methylation patterns could distinguish intracranial meningiomas from metastatic meningiomas. A case report in 2020 showed that DNA methylation clusters distinguished liver metastasis samples from intracranial meningioma samples, indicating that DNA methylation is also a robust method for diagnosing metastatic lesions (117).

**Table 2:** Overview of different classifications of meningiomas in latest studies.

DNA Methylation Classification (MC)	WHO Grade	Possible Biological Group
MC Benign 1 (Mc ben-1)	Grade I/II	immunogenic benign NF2 wild-type hypermetabolic proliferative
MC Benign 2 (Mc ben-2)	Grade I	
MC Benign 3 (Mc ben-3)	Grade I/II	
MC intermediate A (Mc int-A)	Grade I/II	
MC intermediate B (Mc int-B)	Grade II/III	
MC malignant (Mc mal)	Grade II/III	
(115)		(12)

A 2022 study classified meningiomas into three methylation groups, similar to the study by Sahm and co-workers (102), and showed that DNA methylation is more accurate than histopathology in identifying high-risk tumors and is closely correlated with gene expression in meningiomas (118). This study further compared the predictive accuracy of DNA methylation with that of RNA-sequencing and cytogenetics and found a strong concordance between these groups. The authors also demonstrated that both DNA promoter methylation and copy-number variability correlated with differential gene expression (118). Further, a recent study analyzed four types of alterations together, namely DNA somatic copy-number aberrations, DNA somatic point mutations, DNA methylation, and messenger RNA abundance, and found that these could be classified into four groups (M1–4) owing to distinct biology as follows: immunogenic (M1), benign NF2 wild-type (M2), hypermetabolic (M3), and proliferative (M4) (12) **(** **Table 2** **)**. **Table 2** describes the different classifications of meningiomas. From the authors’ perspective, the M2 group might be associated with angiogenesis and vasculature development. Hypermetabolic (MG3) meningiomas are enriched in protein pathways involved in nucleotide and lipid metabolism and could be related to degradation of the extracellular matrix and endothelial proliferation. Moreover, proliferative (MG4) meningiomas are enriched in proteins and genes regulating the cell cycle and proliferation. Distorted DNA methylation processes can be associated with the most aggressive molecular groups (M3–4). Those patients with MG3 and MG4 meningiomas have significantly shorter times to recurrence (log-rank test, P = 5 × 10^−15^) (12). No other studies have discussed the direct association between DNA methylation and biological mechanisms. These data show that DNA methylation has a powerful predictive value. The combination of DNA methylation and other features might be a new direction for identifying high-risk recurrent or progressive meningiomas. In the future, classifications based on more molecular features might be more accurate to predict the prognosis and guide the treatment of meningiomas.

## Conclusions and Perspectives

Here, we reviewed the potential mechanisms underlying recurrent and progressive meningiomas from focused perspectives, specifically the excessive proliferation of tumor cells, neo-angiogenesis, the inhibition of apoptosis, and genetic alterations. We also describe some potential therapeutic targets and prognostic biomarkers for meningiomas from these perspectives. Although we discussed these pathological processes separately, studies have shown that they do not occur in isolation. The histological classification of tumors has shown that those high-risk meningiomas often have the following characteristics: abundant blood vessels, increased nuclear mitosis, increased cell density, loss of tumor inherent structure, blurred basement membrane, and cerebral invasion or metastasis. In the final subsection, we also summarized the chromosomal abnormalities associated with these recurrent or progressive meningiomas, but research on the key biological pathways and their characteristics is still limited. We briefly compared the latest classification of meningiomas based on DNA methylation with the WHO grade and showed that the DNA methylation classification provides a more current prognosis, which requires further confirmation. Because of the complex and subtle changes caused by genetic abnormalities or other undetected factors, the precise mechanism underlying the pathology of meningiomas remains an enigma. Therefore, an in-depth understanding of the development of recurrent and progressive meningiomas is further required to block the disease process and improve the prognosis of the disease.

## Author Contributions

All authors listed have made a substantial, direct, and intellectual contribution to the work, and approved it for publication. WP, JZ and QL were responsible for the conception of the idea, and manuscript preparation; MY and LZ primarily created the figure and tables; PW and JZ prepared and revised the manuscript.

## Funding

This work was supported by grants from the National Natural Science Foundation (81901527) and Natural Science Foundation of Chongqing (cstc2021jcyj-msxmX0862).

## Conflict of Interest

The authors declare that the research was conducted in the absence of any commercial or financial relationships that could be construed as a potential conflict of interest.

## Publisher’s Note

All claims expressed in this article are solely those of the authors and do not necessarily represent those of their affiliated organizations, or those of the publisher, the editors and the reviewers. Any product that may be evaluated in this article, or claim that may be made by its manufacturer, is not guaranteed or endorsed by the publisher.

## Abbreviations

WHO, World Health Organization; FOXM1, Forkhead box protein M1; VEGFR, Vascular endothelial growth factor receptor; MDM2, Murine double minute 2 protein; FAK, Focal adhesion kinase; CDKN2A/B, Cyclin-dependent kinase inhibitor 2A/B; DMD, Dystrophin-encoding and muscular dystrophy-associated; AKT1, v-Akt murine thymoma viral oncogene; HIF-1α, Hypoxia-inducible factor 1α; PI3K, Phosphatidylinositol 3-kinase; mTOR, mammalian Target of rapamycin; CDK, Cyclin-dependent kinase; BAP1, Breast cancer 1-associated protein-1; TERTp, promoter of Telomerase reverse transcriptase; p-CREB, phosphorylated Cyclic-AMP responsive element-binding protein; RAS, Rat sarcoma; PFS, Progression-free survival; KLF4, Krüppel-like factor 4; VEGF, Vascular endothelial growth factor; MC, Methylation classification; PD-1, Programmed death 1; PD-L1, Programmed death-ligand 1.

## References

[1] Preusser M Brastianos P Mawrin C. Advances in Meningioma Genetics: Novel Therapeutic Opportunities. Nat Rev Neurol (2018) 14:106–15. doi: 10.1038/nrneurol.2017.168 29302064

[2] Goldbrunner R Stavrinou P Jenkinson M Sahm F Mawrin C Weber D. EANO Guideline on the Diagnosis and Management of Meningiomas. Neuro-Oncology (2021) 23(11):1821–34. doi: 10.1093/neuonc/noab150 PMC8563316 34181733

[3] Qin C Huang M Pan Y Li Y Long W Liu Q. Brain-Invasive Meningiomas: Molecular Mechanisms and Potential Therapeutic Options. Brain Tumor Pathol (2021) 38:156–72. doi: 10.1007/s10014-021-00399-x 33903981

[4] Shao Z Liu L Zheng Y Tu S Pan Y Yan S. Molecular Mechanism and Approach in Progression of Meningioma. Front Oncol (2020) 10:538845. doi: 10.3389/fonc.2020.538845 33042832 PMC7518150

[5] Brastianos P Galanis E Butowski N Chan J Dunn I Goldbrunner R. Advances in Multidisciplinary Therapy for Meningiomas. Neuro-oncology (2019) 21:i18–31. doi: 10.1093/neuonc/noy136 PMC6347080 30649489

[6] Delgado-López P Cubo-Delgado E González-Bernal J Martín-Alonso J. A Practical Overview on the Molecular Biology of Meningioma. Curr Neurol Neurosci Rep (2020) 20:62. doi: 10.1007/s11910-020-01084-w 33136206

[7] Buerki R Horbinski C Kruser T Horowitz P James C Lukas R. An Overview of Meningiomas. Future Oncol (London England) (2018) 14:2161–77. doi: 10.2217/fon-2018-0006 PMC6123887 30084265

[8] Kshettry V Ostrom Q Kruchko C Al-Mefty O Barnett G Barnholtz-Sloan J. Descriptive Epidemiology of World Health Organization Grades II and III Intracranial Meningiomas in the United States. Neuro-Oncology (2015) 17:1166–73. doi: 10.1093/neuonc/nov069 PMC4490879 26008603

[9] Cordova C Kurz S. Advances in Molecular Classification and Therapeutic Opportunities in Meningiomas. Curr Oncol Rep (2020) 22:84. doi: 10.1007/s11912-020-00937-4 32617743

[10] Quddusi A Virani Q Shamim M. Factors Affecting Post-Operative Recurrence or Growth of Meningiomas, Other Than Histological Grade and Extent of Resection. JPMA J Pakistan Med Assoc (2019) 69:1570–1. 31622321

[11] Wilson T Huang L Ramanathan D Lopez-Gonzalez M Pillai P De Los Reyes K. Review of Atypical and Anaplastic Meningiomas: Classification, Molecular Biology, and Management. Front Oncol (2020) 10:565582. doi: 10.3389/fonc.2020.565582 33330036 PMC7714950

[12] Nassiri F Liu J Patil V Mamatjan Y Wang J Hugh-White R. A Clinically Applicable Integrative Molecular Classification of Meningiomas. Nature (2021) 597:119–25. doi: 10.1038/s41586-021-03850-3 PMC11604310 34433969

[13] Driver J Hoffman S Tavakol S Woodward E Maury E Bhave V. A Molecularly Integrated Grade for Meningioma. Neuro-Oncology (2021) 24(5):796–808. doi: 10.1093/neuonc/noab213 PMC9071299 34508644

[14] Alvarez-Fernández M Medema R. Novel Functions of FoxM1: From Molecular Mechanisms to Cancer Therapy. Front Oncol (2013) 3:30. doi: 10.3389/fonc.2013.00030 23467617 PMC3588610

[15] Winther T Torp S. DNA Topoisomerase Iiα and Mitosin Expression Predict Meningioma Recurrence Better Than Histopathological Grade and MIB-1 After Initial Surgery. PloS One (2017) 12:e0172316. doi: 10.1371/journal.pone.0172316 28301542 PMC5354255

[16] Juratli T McCabe D Nayyar N Williams E Silverman I Tummala S. DMD Genomic Deletions Characterize a Subset of Progressive/Higher-Grade Meningiomas With Poor Outcome. Acta Neuropathologica (2018) 136:779–92. doi: 10.1007/s00401-018-1899-7 30123936

[17] Lee Y Lee Y. Molecular Characteristics of Meningiomas. J Pathol Trans Med (2020) 54:45–63. doi: 10.4132/jptm.2019.11.05 PMC6986967 31964111

[18] Ohta S Taniguchi T Sato N Hamada M Taniguchi H Rappsilber J. Quantitative Proteomics of the Mitotic Chromosome Scaffold Reveals the Association of BAZ1B With Chromosomal Axes. Mol Cell Proteomics MCP (2019) 18:169–81. doi: 10.1074/mcp.RA118.000923 PMC6356081 30266865

[19] Kalinichenko V Major M Wang X Petrovic V Kuechle J Yoder H. Foxm1b Transcription Factor is Essential for Development of Hepatocellular Carcinomas and is Negatively Regulated by the P19arf Tumor Suppressor. Genes Dev (2004) 18:830–50. doi: 10.1101/gad.1200704 PMC387422 15082532

[20] Kalin T Wang I Ackerson T Major M Detrisac C Kalinichenko V. Increased Levels of the FoxM1 Transcription Factor Accelerate Development and Progression of Prostate Carcinomas in Both TRAMP and LADY Transgenic Mice. Cancer Res (2006) 66:1712–20. doi: 10.1158/0008-5472.Can-05-3138 PMC1363687 16452231

[21] Liu M Dai B Kang S Ban K Huang F Lang F. FoxM1B Is Overexpressed in Human Glioblastomas and Critically Regulates the Tumorigenicity of Glioma Cells. Cancer Res (2006) 66:3593–602. doi: 10.1158/0008-5472.Can-05-2912 16585184

[22] Teh M Wong S Neill G Ghali L Philpott M Quinn A. FOXM1 Is a Downstream Target of Gli1 in Basal Cell Carcinomas. Cancer Res (2002) 62:4773–80. 12183437

[23] Vasudevan H Braunstein S Phillips J Pekmezci M Tomlin B Wu A. Comprehensive Molecular Profiling Identifies FOXM1 as a Key Transcription Factor for Meningioma Proliferation. Cell Rep (2018) 22:3672–83. doi: 10.1016/j.celrep.2018.03.013 PMC8204688 29590631

[24] Yamazaki S Ohka F Hirano M Shiraki Y Motomura K Tanahashi K. Newly Established Patient-Derived Organoid Model of Intracranial Meningioma. Neuro-Oncology (2021) 23(11):1936–48. doi: 10.1093/neuonc/noab155 PMC8563327 34214169

[25] Brastianos P Horowitz P Santagata S Jones R McKenna A Getz G. Genomic Sequencing of Meningiomas Identifies Oncogenic SMO and AKT1 Mutations. Nat Genet (2013) 45:285–9. doi: 10.1038/ng.2526 PMC3739288 23334667

[26] Carpten J Faber A Horn C Donoho G Briggs S Robbins C. A Transforming Mutation in the Pleckstrin Homology Domain of AKT1 in Cancer. Nature (2007) 448:439–44. doi: 10.1038/nature05933 17611497

[27] Barresi V Lionti S La Rocca L Caliri S Caffo M. High p-mTOR Expression is Associated With Recurrence and Shorter Disease-Free Survival in Atypical Meningiomas. Neuropathology (2019) 39:22–9. doi: 10.1111/neup.12524 30511495

[28] Yesilöz Ü Kirches E Hartmann C Scholz J Kropf S Sahm F. Frequent AKT1E17K Mutations in Skull Base Meningiomas Are Associated With mTOR and ERK1/2 Activation and Reduced Time to Tumor Recurrence. Neuro-oncology (2017) 19:1088–96. doi: 10.1093/neuonc/nox018 PMC5570238 28482067

[29] Clark V Erson-Omay E Serin A Yin J Cotney J Ozduman K. Genomic Analysis of Non-NF2 Meningiomas Reveals Mutations in TRAF7, KLF4, AKT1, and SMO. Sci (New York N.Y.) (2013) 339:1077–80. doi: 10.1126/science.1233009 PMC4808587 23348505

[30] Ng J Curran T. The Hedgehog's Tale: Developing Strategies for Targeting Cancer. Nat Rev Cancer (2011) 11:493–501. doi: 10.1038/nrc3079 21614026 PMC3576812

[31] Wang X Gong Y Wang D Xie Q Zheng M Zhou Y. Analysis of Gene Expression Profiling in Meningioma: Deregulated Signaling Pathways Associated With Meningioma and EGFL6 Overexpression in Benign Meningioma Tissue and Serum. PloS One (2012) 7:e52707. doi: 10.1371/journal.pone.0052707 23285163 PMC3532066

[32] Kim L. A Narrative Review of Targeted Therapies in Meningioma. Chin Clin Oncol (2020) 9:76. doi: 10.21037/cco-2020-mbt-01 33353364

[33] Maier A Stenman A Svahn F Mirian C Bartek J Juhler M. TERT Promoter Mutations in Primary and Secondary WHO Grade III Meningioma. Brain Pathol (Zurich Switzerland) (2021) 31:61–9. doi: 10.1111/bpa.12892 PMC8018144 32805769

[34] Wei Y Xia W Zhang Z Liu J Wang H Adsay N. Loss of Trimethylation at Lysine 27 of Histone H3 Is a Predictor of Poor Outcome in Breast, Ovarian, and Pancreatic Cancers. Mol Carcinog (2008) 47:701–6. doi: 10.1002/mc.20413 PMC2580832 18176935

[35] Dasanu C Alvarez-Argote J Limonadi F Codreanu I. Bevacizumab in Refractory Higher-Grade and Atypical Meningioma: The Current State of Affairs. Expert Opin Biol Ther (2019) 19:99–104. doi: 10.1080/14712598.2019.1559292 30556741

[36] Franke A Skelton W Woody L Bregy A Shah A Vakharia K. Role of Bevacizumab for Treatment-Refractory Meningiomas: A Systematic Analysis and Literature Review. Surg Neurol Int (2018) 9:133. doi: 10.4103/sni.sni_264_17 30090665 PMC6057170

[37] Brastianos P Kim A Giobbie-Hurder A Lee E Wang N Eichler A. Phase 2 Study of Pembrolizumab in Patients With Recurrent and Residual High-Grade Meningiomas. Nat Commun (2022) 13:1325. doi: 10.1038/s41467-022-29052-7 35289329 PMC8921328

[38] Moussalem C Massaad E Minassian G Ftouni L Bsat S Houshiemy M. Meningioma Genomics: A Therapeutic Challenge for Clinicians. J Integr Neurosci (2021) 20:463–9. doi: 10.31083/j.jin2002049 34258948

[39] Graillon T Sanson M Campello C Idbaih A Peyre M Peyrière H. Everolimus and Octreotide for Patients With Recurrent Meningioma: Results From the Phase II CEVOREM Trial. Clin Cancer Res (2020) 26:552–7. doi: 10.1158/1078-0432.Ccr-19-2109 31969329

[40] Nassiri F Mamatjan Y Suppiah S Badhiwala J Mansouri S Karimi S. DNA Methylation Profiling to Predict Recurrence Risk in Meningioma: Development and Validation of a Nomogram to Optimize Clinical Management. Neuro-oncology (2019) 21:901–10. doi: 10.1093/neuonc/noz061 PMC6620635 31158293

[41] Boström J Meyer-Puttlitz B Wolter M Blaschke B Weber R Lichter P. Alterations of the Tumor Suppressor Genes CDKN2A (P16(INK4a)), P14(ARF), CDKN2B (P15(INK4b)), and CDKN2C (P18(INK4c)) in Atypical and Anaplastic Meningiomas. Am J Pathol (2001) 159:661–9. doi: 10.1016/s0002-9440(10)61737-3 PMC1850553 11485924

[42] Perry A Banerjee R Lohse C Kleinschmidt-DeMasters B Scheithauer B. A Role for Chromosome 9p21 Deletions in the Malignant Progression of Meningiomas and the Prognosis of Anaplastic Meningiomas. Brain Pathol (Zurich Switzerland) (2002) 12:183–90. doi: 10.1111/j.1750-3639.2002.tb00433.x PMC8095834 11958372

[43] Trott G Pereira-Lima J Leães C Ferreira N Barbosa-Coutinho L Oliveira M. Abundant Immunohistochemical Expression of Dopamine D2 Receptor and P53 Protein in Meningiomas: Follow-Up, Relation to Gender, Age, Tumor Grade, and Recurrence. Braz J Med Biol Res = Rev Bras Pesquisas Medicas e Biologicas (2015) 48:415–9. doi: 10.1590/1414-431x20144163 PMC4445664 25742638

[44] Hansen S Hart M Busi S Parker T Goerndt A Jones K. Fischer-344 Tp53-Knockout Rats Exhibit a High Rate of Bone and Brain Neoplasia With Frequent Metastasis. Dis Models Mech (2016) 9:1139–46. doi: 10.1242/dmm.025767 PMC5087826 27528400

[45] Pavelin S Becic K Forempoher G Mrklic I Pogorelic Z Titlic M. Expression of Ki-67 and P53 in Meningiomas. Neoplasma (2013) 60:480–5. doi: 10.4149/neo_2013_062 23790165

[46] Ervasti J. Dystrophin, its Interactions With Other Proteins, and Implications for Muscular Dystrophy. Biochim Biophys Acta (2007) 1772:108–17. doi: 10.1016/j.bbadis.2006.05.010 16829057

[47] Prins K Humston J Mehta A Tate V Ralston E Ervasti J. Dystrophin Is a Microtubule-Associated Protein. J Cell Biol (2009) 186:363–9. doi: 10.1083/jcb.200905048 PMC2728405 19651889

[48] Reuss D Piro R Jones D Simon M Ketter R Kool M. Secretory Meningiomas Are Defined by Combined KLF4 K409Q and TRAF7 Mutations. Acta Neuropathologica (2013) 125:351–8. doi: 10.1007/s00401-013-1093-x 23404370

[49] Shankar G Abedalthagafi M Vaubel R Merrill P Nayyar N Gill C. Germline and Somatic BAP1 Mutations in High-Grade Rhabdoid Meningiomas. Neuro-oncology (2017) 19:535–45. doi: 10.1093/neuonc/now235 PMC5464371 28170043

[50] Collord G Tarpey P Kurbatova N Martincorena I Moran S Castro M. An Integrated Genomic Analysis of Anaplastic Meningioma Identifies Prognostic Molecular Signatures. Sci Rep (2018) 8:13537. doi: 10.1038/s41598-018-31659-0 30202034 PMC6131140

[51] Spiegl-Kreinecker S Lötsch D Neumayer K Kastler L Gojo J Pirker C. TERT Promoter Mutations Are Associated With Poor Prognosis and Cell Immortalization in Meningioma. Neuro-oncology (2018) 20:1584–93. doi: 10.1093/neuonc/noy104 PMC6231195 30010853

[52] Juratli T Thiede C Koerner M Tummala S Daubner D Shankar G. TERTIntratumoral Heterogeneity and Promoter Mutations in Progressive/Higher-Grade Meningiomas. Oncotarget (2017) 8:109228–37. doi: 10.18632/oncotarget.22650 PMC5752516 29312603

[53] Mellai M Porrini Prandini O Mustaccia A Fogazzi V Allesina M Krengli M. Human TERT Promoter Mutations in Atypical and Anaplastic Meningiomas. Diagnostics (Basel Switzerland) (2021) 11:1624. doi: 10.3390/diagnostics11091624 PMC8469948 34573966

[54] Goutagny S Nault J Mallet M Henin D Rossi J Kalamarides M. High Incidence of Activating TERT Promoter Mutations in Meningiomas Undergoing Malignant Progression. Brain Pathol (Zurich Switzerland) (2014) 24:184–9. doi: 10.1111/bpa.12110 PMC8029399 24261697

[55] Deng J Sun S Chen J Wang D Cheng H Chen H. TERT Alterations Predict Tumor Progression in High-Grade Meningiomas Following Adjuvant Radiotherapy. Front Oncol (2021) 11:747592. doi: 10.3389/fonc.2021.747592 34778063 PMC8586415

[56] Katz L Hielscher T Liechty B Silverman J Zagzag D Sen R. Loss of Histone H3K27me3 Identifies a Subset of Meningiomas With Increased Risk of Recurrence. Acta Neuropathologica (2018) 135:955–63. doi: 10.1007/s00401-018-1844-9 29627952

[57] Jung M Kim S Lim K Bae J Park C Choi S. The Substantial Loss of H3K27me3 can Stratify Risk in Grade 2, But Not in Grade 3 Meningioma. Hum Pathol (2021) 115:96–103. doi: 10.1016/j.humpath.2021.06.005 34186055

[58] Nassiri F Wang J Singh O Karimi S Dalcourt T Ijad N. Loss of H3K27me3 in Meningiomas. Neuro-oncology (2021) 23:1282–91. doi: 10.1093/neuonc/noab036 PMC8328029 33970242

[59] Hess K Spille D Adeli A Sporns P Zitta K Hummitzsch L. Occurrence of Fibrotic Tumor Vessels in Grade I Meningiomas Is Strongly Associated With Vessel Density, Expression of VEGF, PlGF, IGFBP-3 and Tumor Recurrence. Cancers (2020) 12:3075. doi: 10.3390/cancers12103075 PMC7593950 33096816

[60] Bernatz S Monden D Gessler F Radic T Hattingen E Senft C. Influence of VEGF-A, VEGFR-1-3, and Neuropilin 1-2 on Progression-Free: And Overall Survival in WHO Grade II and III Meningioma Patients. J Mol Histol (2021) 52:233–43. doi: 10.1007/s10735-020-09940-2 PMC8012320 33528717

[61] Nassehi D Dyrbye H Andresen M Thomsen C Juhler M Laursen H. Vascular Endothelial Growth Factor A Protein Level and Gene Expression in Intracranial Meningiomas With Brain Edema. APMIS (2011) 119:831–43. doi: 10.1111/j.1600-0463.2011.02764.x 22085359

[62] Xiao Z Chen X Pan Q Yang Q Li K. Expression of Nestin, CD133 and Sox2 in Meningiomas. Turkish Neurosurg (2018) 28:910–4. doi: 10.5137/1019-5149.Jtn.21234-17.2 29368320

[63] Nassehi D. Intracranial Meningiomas, the VEGF-A Pathway, and Peritumoral Brain Oedema. Danish Med J (2013) 60:B4626. 23651727

[64] Dasanu C Samara Y Codreanu I Limonadi F Hamid O Alvarez-Argote J. Systemic Therapy for Relapsed/Refractory Meningioma: Is There Potential for Antiangiogenic Agents? J Oncol Pharm Pract (2019) 25:638–47. doi: 10.1177/1078155218799850 30253729

[65] Barresi V Branca G Caffo M Tuccari G. P-CREB Expression in Human Meningiomas: Correlation With Angiogenesis and Recurrence Risk. J Neuro-Oncol (2015) 122:87–95. doi: 10.1007/s11060-014-1706-9 25563814

[66] Barresi V Mondello S Branca G Rajan T Vitarelli E Tuccari G. P-CREB Expression in Human Gliomas: Potential Use in the Differential Diagnosis Between Astrocytoma and Oligodendroglioma. Hum Pathol (2015) 46:231–8. doi: 10.1016/j.humpath.2014.10.011 25476123

[67] Khan I Baeesa S Bangash M Schulten H Alghamdi F Qashqari H. Pleomorphism and Drug Resistant Cancer Stem Cells Are Characteristic of Aggressive Primary Meningioma Cell Lines. Cancer Cell Int (2017) 17:72. doi: 10.1186/s12935-017-0441-7 28736504 PMC5521079

[68] Ager E Neo J Christophi C. The Renin-Angiotensin System and Malignancy. Carcinogenesis (2008) 29:1675–84. doi: 10.1093/carcin/bgn171 18632755

[69] Tirakotai W Mennel H Celik I Hellwig D Bertalanffy H Riegel T. Secretory Meningioma: Immunohistochemical Findings and Evaluation of Mast Cell Infiltration. Neurosurgical Rev (2006) 29:41–8. doi: 10.1007/s10143-005-0402-9 16010579

[70] Mohammad R Muqbil I Lowe L Yedjou C Hsu H Lin L. Broad Targeting of Resistance to Apoptosis in Cancer. Semin Cancer Biol (2015) 35:S78–103. doi: 10.1016/j.semcancer.2015.03.001 25936818 PMC4720504

[71] Che Q Huang T Zhang Y Qian X. Effect of miR-124 on Neuronal Apoptosis in Rats With Cerebral Infarction Through Wnt/β-Catenin Signaling Pathway. Eur Rev Med Pharmacol Sci (2019) 23:6657–64. doi: 10.26355/eurrev_201908_18556 31378908

[72] Ruan W Hu J Zhou H Li Y Xu C Luo Y. Intranasal Wnt-3a Alleviates Neuronal Apoptosis in Early Brain Injury Post Subarachnoid Hemorrhage *via* the Regulation of Wnt Target PPAN Mediated by the Moonlighting Role of Aldolase C. Neurochem Int (2020) 134:104656. doi: 10.1016/j.neuint.2019.104656 31899197

[73] Gao K Shen Z Yuan Y Han D Song C Guo Y. Simvastatin Inhibits Neural Cell Apoptosis and Promotes Locomotor Recovery *via* Activation of Wnt/β-Catenin Signaling Pathway After Spinal Cord Injury. J Neurochem (2016) 138:139–49. doi: 10.1111/jnc.13382 PMC5089634 26443048

[74] Li C Jiao G Wu W Wang H Ren S Zhang L. Exosomes From Bone Marrow Mesenchymal Stem Cells Inhibit Neuronal Apoptosis and Promote Motor Function Recovery *via* the Wnt/β-Catenin Signaling Pathway. Cell Transplant (2019) 28:1373–83. doi: 10.1177/0963689719870999 PMC6802144 31423807

[75] Tian X Hou W Fang Y Fan J Tong H Bai S. XAV939, a Tankyrase 1 Inhibitior, Promotes Cell Apoptosis in Neuroblastoma Cell Lines by Inhibiting Wnt/β-Catenin Signaling Pathway. J Exp Clin Cancer Res CR (2013) 32:100. doi: 10.1186/1756-9966-32-100 24308762 PMC3866601

[76] Li Q Shen K Zhao Y Ma C Liu J Ma J. MiR-92b Inhibitor Promoted Glioma Cell Apoptosis *via* Targeting DKK3 and Blocking the Wnt/beta-Catenin Signaling Pathway. J Trans Med (2013) 11:302. doi: 10.1186/1479-5876-11-302 PMC4028874 24325785

[77] Pećina-Šlaus N Kafka A Lechpammer M. Molecular Genetics of Intracranial Meningiomas With Emphasis on Canonical Wnt Signalling. Cancers (2016) 8. doi: 10.3390/cancers8070067 PMC4963809 27429002

[78] Shi Z Fan Z Chen Y Xie X Jiang W Wang W. Ferroptosis in Carcinoma: Regulatory Mechanisms and New Method for Cancer Therapy. OncoTargets Ther (2019) 12:11291–304. doi: 10.2147/ott.S232852 PMC6927606 31908494

[79] Zhang Y Yu R Li Q Li Y Xuan T Cao S. SNHG1/miR-556-5p/TCF12 Feedback Loop Enhances the Tumorigenesis of Meningioma Through Wnt Signaling Pathway. J Cell Biochem (2020) 121:1880–9. doi: 10.1002/jcb.29423 31692066

[80] Sharma S Ray S Mukherjee S Moiyadi A Sridhar E Srivastava S. Multipronged Quantitative Proteomic Analyses Indicate Modulation of Various Signal Transduction Pathways in Human Meningiomas. Proteomics (2015) 15:394–407. doi: 10.1002/pmic.201400328 25413884

[81] Das A Miller R Lee P Holden C Lindhorst S Jaboin J. A Novel Component From Citrus, Ginger, and Mushroom Family Exhibits Antitumor Activity on Human Meningioma Cells Through Suppressing the Wnt/β-Catenin Signaling Pathway. Tumour Biol J Int Soc Oncodevelopmental Biol Med (2015) 36:7027–34. doi: 10.1007/s13277-015-3388-0 PMC9499733 25864108

[82] Kanno H Nishihara H Wang L Yuzawa S Kobayashi H Tsuda M. Expression of CD163 Prevents Apoptosis Through the Production of Granulocyte Colony-Stimulating Factor in Meningioma. Neuro-Oncology (2013) 15:853–64. doi: 10.1093/neuonc/not028 PMC3688010 23539121

[83] Tsukita S Furuse M. Claudin-Based Barrier in Simple and Stratified Cellular Sheets. Curr Opin Cell Biol (2002) 14:531–6. doi: 10.1016/s0955-0674(02)00362-9 12231346

[84] Liu Y Jin X Li Y Ruan Y Lu Y Yang M. DNA Methylation of Claudin-6 Promotes Breast Cancer Cell Migration and Invasion by Recruiting MeCP2 and Deacetylating H3Ac and H4Ac. J Exp Clin Cancer Res CR (2016) 35:120. doi: 10.1186/s13046-016-0396-x 27461117 PMC4962420

[85] Guo Y Lin D Zhang M Zhang X Li Y Yang R. CLDN6-Induced Apoptosis *via* Regulating ASK1-P38/JNK Signaling in Breast Cancer MCF-7 Cells. Int J Oncol (2016) 48:2435–44. doi: 10.3892/ijo.2016.3469 27035750

[86] Torres-Martínez A Gallardo-Vera J Lara-Holguin A Montaño L Rendón-Huerta E. Claudin-6 Enhances Cell Invasiveness Through Claudin-1 in AGS Human Adenocarcinoma Gastric Cancer Cells. Exp Cell Res (2017) 350:226–35. doi: 10.1016/j.yexcr.2016.11.025 27914788

[87] Wang Q Zhang Y Zhang T Han Z Shan L. Low Claudin-6 Expression Correlates With Poor Prognosis in Patients With Non-Small Cell Lung Cancer. OncoTargets Ther (2015) 8:1971–7. doi: 10.2147/ott.S85478 PMC4527519 26261421

[88] Yang A Yang X Wang J Wang X Wu H Fan L. Effects of the Tight Junction Protein CLDN6 on Cell Migration and Invasion in High-Grade Meningioma. World Neurosurg (2021) 151:e208-e216. doi: 10.1016/j.wneu.2021.04.005 33862296

[89] Takashima A Faller D. Targeting the RAS Oncogene. Expert Opin Ther Targets (2013) 17:507–31. doi: 10.1517/14728222.2013.764990 PMC3804031 23360111

[90] Garzon R Pichiorri F Palumbo T Visentini M Aqeilan R Cimmino A. MicroRNA Gene Expression During Retinoic Acid-Induced Differentiation of Human Acute Promyelocytic Leukemia. Oncogene (2007) 26:4148–57. doi: 10.1038/sj.onc.1210186 17260024

[91] Ramberg H Alshbib A Berge V Svindland A Taskén K. Regulation of PBX3 Expression by Androgen and Let-7d in Prostate Cancer. Mol Cancer (2011) 10:50. doi: 10.1186/1476-4598-10-50 21548940 PMC3112428

[92] Masood F Khan W Uddin R. Computational-Based Identification and Analysis of Globally Expressed Differential Genes in High-Grade Serous Ovarian Carcinoma Cell Lines. Comput Biol Chem (2020) 88:107333. doi: 10.1016/j.compbiolchem.2020.107333 32738584

[93] Li H Zhao J. Let-7d Suppresses Proliferation and Invasion and Promotes Apoptosis of Meningioma by Targeting AEG-1. OncoTargets Ther (2017) 10:4895–904. doi: 10.2147/ott.S141008 PMC5640403 29070952

[94] Castilla-Vallmanya L Selmer K Dimartino C Rabionet R Blanco-Sánchez B Yang S. Phenotypic Spectrum and Transcriptomic Profile Associated With Germline Variants in TRAF7. Genet Med (2020) 22:1215–26. doi: 10.1038/s41436-020-0792-7 PMC8093014 32376980

[95] Birzu C Peyre M Sahm F. Molecular Alterations in Meningioma: Prognostic and Therapeutic Perspectives. Curr Opin Oncol (2020) 32:613–22. doi: 10.1097/cco.0000000000000687 32890025

[96] Venur V Santagata S Galanis E Brastianos P. New Molecular Targets in Meningiomas: The Present and the Future. Curr Opin Neurol (2018) 31:740–6. doi: 10.1097/wco.0000000000000615 30379704

[97] Bi W Prabhu V Dunn I. High-Grade Meningiomas: Biology and Implications. Neurosurgical Focus (2018) 44:E2. doi: 10.3171/2017.12.Focus17756 29606053

[98] Abedalthagafi M Bi W Aizer A Merrill P Brewster R Agarwalla P. Oncogenic PI3K Mutations Are as Common as AKT1 and SMO Mutations in Meningioma. Neuro-Oncology (2016) 18:649–55. doi: 10.1093/neuonc/nov316 PMC4827048 26826201

[99] Yeung J Yaghoobi V Miyagishima D Vesely M Zhang T Badri T. Targeting the CSF1/CSF1R Axis Is a Potential Treatment Strategy for Malignant Meningiomas. Neuro-Oncology (2021) 23:1922–35. doi: 10.1093/neuonc/noab075 PMC8563319 33914067

[100] Terabe M Wu J. Rethinking Immunotherapy in Meningiomas. Neuro-Oncology (2021) 23:1812–13. doi: 10.1093/neuonc/noab168 PMC8563318 34244788

[101] Chamberlain M Glantz M. Interferon-Alpha for Recurrent World Health Organization Grade 1 Intracranial Meningiomas. Cancer (2008) 113:2146–51. doi: 10.1002/cncr.23803 18756531

[102] Parada C Osbun J Kaur S Yakkioui Y Shi M Pan C. Kinome and Phosphoproteome of High-Grade Meningiomas Reveal AKAP12 as a Central Regulator of Aggressiveness and its Possible Role in Progression. Sci Rep (2018) 8:2098. doi: 10.1038/s41598-018-19308-y 29391485 PMC5794791

[103] Riemenschneider M Perry A Reifenberger G. Histological Classification and Molecular Genetics of Meningiomas. Lancet Neurol (2006) 5:1045–54. doi: 10.1016/s1474-4422(06)70625-1 17110285

[104] Keppler-Noreuil K Baker E Sapp J Lindhurst M Biesecker L. Somatic AKT1 Mutations Cause Meningiomas Colocalizing With a Characteristic Pattern of Cranial Hyperostosis. Am J Med Genet Part A (2016) 170:2605–10. doi: 10.1002/ajmg.a.37737 PMC5580816 27550858

[105] Wang J Lv P. Chrysophanol Inhibits the Osteoglycin/mTOR and Activats NF2 Signaling Pathways to Reduce Viability and Proliferation of Malignant Meningioma Cells. Bioengineered (2021) 12:755–62. doi: 10.1080/21655979.2021.1885864 PMC8291820 33622177

[106] Harmancı A Youngblood M Clark V Coşkun S Henegariu O Duran D. Integrated Genomic Analyses of *De Novo* Pathways Underlying Atypical Meningiomas. Nat Commun (2017) 8:14433. doi: 10.1038/ncomms14433 28195122 PMC5316884

[107] Chang L Oblinger J Smith A Ferrer M Angus S Hawley E. Brigatinib Causes Tumor Shrinkage in Both NF2-Deficient Meningioma and Schwannoma Through Inhibition of Multiple Tyrosine Kinases But Not ALK. PloS One (2021) 16:e0252048. doi: 10.1371/journal.pone.0252048 34264955 PMC8282008

[108] Brastianos PK Twohy E Gerstner ER Kaufmann TJ Iafrate AJ Jeyapalan SA. Alliance A071401: Phase II Trial of FAK Inhibition in Meningiomas With Somatic NF2 Mutations. J Clin Oncol (2020) 38:2502–2. doi: 10.1200/JCO.2020.38.15_suppl.2502 PMC9870228 36288512

[109] Patel A Wan Y Al-Ouran R Revelli J Cardenas M Oneissi M. Molecular Profiling Predicts Meningioma Recurrence and Reveals Loss of DREAM Complex Repression in Aggressive Tumors. Proc Natl Acad Sci USA (2019) 116:21715–26. doi: 10.1073/pnas.1912858116 PMC6815170 31591222

[110] Lamszus K. Meningioma Pathology, Genetics, and Biology. J Neuropathol Exp Neurol (2004) 63:275–86. doi: 10.1093/jnen/63.4.275 15099018

[111] Och W Szmuda T Kulbacki K Witek K Sikorska B Zakrzewska M. The Correlation of Clinical and Chromosomal Alterations of Benign Meningiomas and Their Recurrences. Neurologia i Neurochirurgia Polska (2016) 50:395–402. doi: 10.1016/j.pjnns.2016.07.001 27480481

[112] Och W Szmuda T Sikorska B Springer J Jaskólski D Zakrzewska M. Recurrence-Associated Chromosomal Anomalies in Meningiomas: Single-Institution Study and a Systematic Review With Meta-Analysis. Neurologia i Neurochirurgia Polska (2016) 50:439–48. doi: 10.1016/j.pjnns.2016.08.003 27575681

[113] Ogasawara C Philbrick B Adamson D. Meningioma: A Review of Epidemiology, Pathology, Diagnosis, Treatment, and Future Directions. Biomedicines (2021) 9:319. doi: 10.3390/biomedicines9030319 33801089 PMC8004084

[114] Aizer A Abedalthagafi M Bi W Horvath M Arvold N Al-Mefty O. A Prognostic Cytogenetic Scoring System to Guide the Adjuvant Management of Patients With Atypical Meningioma. Neuro-Oncology (2016) 18:269–74. doi: 10.1093/neuonc/nov177 PMC4724184 26323607

[115] Sahm F Schrimpf D Stichel D Jones D Hielscher T Schefzyk S. DNA Methylation-Based Classification and Grading System for Meningioma: A Multicentre, Retrospective Analysis. Lancet Oncol (2017) 18:682–94. doi: 10.1016/s1470-2045(17)30155-9 28314689

[116] Youngblood M Miyagishima D Jin L Gupte T Li C Duran D. Associations of Meningioma Molecular Subgroup and Tumor Recurrence. Neuro-oncology (2021) 23:783–94. doi: 10.1093/neuonc/noaa226 PMC8099468 33068421

[117] Vasudevan H Castro M Lee J Villanueva-Meyer J Bush N McDermott M. DNA Methylation Profiling Demonstrates Superior Diagnostic Classification to RNA-Sequencing in a Case of Metastatic Meningioma. Acta Neuropathologica Commun (2020) 8:82. doi: 10.1186/s40478-020-00952-3 PMC7285578 32517746

[118] Bayley J Hadley C Harmanci A Harmanci A Klisch T Patel A. Multiple Approaches Converge on Three Biological Subtypes of Meningioma and Extract New Insights From Published Studies. Sci Adv (2022) 8:eabm6247. doi: 10.1126/sciadv.abm6247 35108039 PMC11313601

